# Can nursing contribute to reducing mortality from land transport accidents?

**DOI:** 10.1590/0034-7167.2024770401

**Published:** 2024-07-08

**Authors:** Rogério Silva Lima, Ariane Aparecida Rodrigues

**Affiliations:** 1 Universidade Federal de Alfenas. Alfenas, Minas Gerais, Brazil

Morbidity and mortality from external causes, especially those resulting from land transport accidents (LTA), persist as a global challenge, given that they account for around 2 million lives lost annually, impacting various segments of society, especially in health services and systems^(1)^.

Evidence indicates that LTA are preventable, although historically they have been considered inevitable. However, isolated actions are commonly not effective and require intersectoral measures, with convergence of efforts from sectors such as urban, financial, legal and health planning^(1)^.

In the Brazilian context, the scenario is no different. Since the 1970s, the country has faced increasing mortality rates due to factors such as an increase in the motorcycle fleet, precarious road network, with maintenance and signage problems, excessive speed, and persistent infractions, such as driving under the influence of alcohol^(2)^.

Faced with this reality, the United Nations (UN) set the goal, included in the third Sustainable Development Goal (SDG), to reduce deaths and injuries from traffic accidents by half by 2020^(3)^.

Despite initiatives to mitigate the effects of LTA, Brazil needs to move forward in implementing actions aimed at preventing injuries, as well as providing adequate support for assistance and recovery of victims.

This challenge demands a multifaceted approach, considering regional inequalities characteristic of a nation of continental proportions^(4)^. This implies the need for a careful understanding of the factors intrinsic to risk behavior in traffic^(5)^. In fact, it is not possible to propose simple solutions to such complex problems; it is necessary to consider a variety of resources to achieve systemic solutions.

This overview awakens reflections on nursing potential to contribute to reduce mortality related to LTA when analyzing the interface between the health field and the problem at hand.

It is considered that nursing, among health professions, has the greatest capillarity, and its workforce is a critical component for the functioning of health care services and systems^(6)^. From this perspective, its actions are fundamental for intersectoral integration and can represent a valuable resource for advances, particularly in LTA primary and tertiary prevention.

In this regard, within the scope of primary prevention, nursing actions in the field of school education, resulting from a partnership between Primary Health Care and the education sector, can be a tool to encourage new learning that translates into safer traffic behaviors, particularly in relation to children.

Regarding tertiary prevention, i.e., in the initial care for traffic victims, nursing also stands out as an essential human resource. Professionals in the area are included in all segments of the Emergency Care Network (RAU), ranging from Family Health Strategies (FHS) and Mobile Emergency Care Service (SAMU-192), with its Basic, Intermediate (in some locations) and Advanced Life Support Units, to emergency hospital doors^(7)^. Therefore, it can be inferred that initial care for LTA victims is sometimes dependent on the nursing workforce, especially in difficult to access locations, which in Brazil is aggravated by geographic and population differences.

It is considered, therefore, that if reduced mortality due to LTA is inseparable from the quality of initial care for victims and, in this care, the nursing team is fundamental, consequently the quality of worker training is a crucial point in improving indicators.

It is not intended to state that such a challenging goal can be achieved solely with educational strategies, but we cannot lose sight of the fact that the expected improvements necessarily involve this axis of actions, which, for the area, implies recognizing the need for advances in emergency nursing knowledge and practices, in addition to micro and macropolitical efforts to guarantee the adequate preparation of future professionals and those who already work in the field.
