# Supplementary material for: Can nursing contribute to reducing mortality from land transport accidents?
Source: Rev Bras Enferm. 2024 Jul 8;77(4):e770401. doi: 10.1590/0034-7167.2024770401 (PMC11253832; doi:10.1590/0034-7167.2024770401)
Supplement: Translation

# Translation: ¿Puede la enfermería contribuir a reducir la mortalidad por accidentes de transporte terrestre?

**DOI:** 10.1590/0034-7167.2024770401es

**Authors:** Rogério Silva Lima, Ariane Aparecida Rodrigues

**Affiliations:** 1 Universidade Federal de Alfenas. Alfenas, Minas Gerais, Brasil

La morbilidad y la mortalidad por causas externas, especialmente las resultantes de accidentes de transporte terrestre (ATT), persisten como un desafío global, dado que es responsable de alrededor de 2 millones de vidas perdidas anualmente, impactando a diversos segmentos de la sociedad, especialmente en los servicios y sistemas de salud^(1)^.

La evidencia indica que los ATT se pueden prevenir, aunque histórica-mente se han considerado inevitables. Sin embargo, las acciones aisladas comúnmente no son efectivas y requieren medidas intersectoriales, con convergencia de esfuerzos de sectores como la planificación urbana, financiera, legal y de salud^(2)^.

En el contexto brasileño, el escenario no es diferente. Desde la década de 1970, el país ha enfrentado tasas de mortalidad crecientes debido a factores como aumento del parque de motos, red viaria precaria, con problemas de mantenimiento y señalización, exceso de velocidad e infracciones persistentes, como conducir bajo los efectos del alcohol^(2)^.

Ante esta realidad, la Organización de las Naciones Unidas (ONU) se fijó la meta, incluida en el tercer Objetivo de Desarrollo Sostenible (ODS), de reducir a la mitad las muertes y lesiones por accidentes de tráfico para 2020^(3)^.

A pesar de las iniciativas para mitigar los efectos del ATT, Brasil necesita avanzar en la implementación de acciones destinadas a prevenir lesiones, así como brindar apoyo adecuado para la asistencia y recuperación de las víctimas.

Este desafío exige un enfoque multifacético, considerando las desigualdades regionales características de una nación de proporciones continentales^(4)^. Esto implica la necesidad de una comprensión cuidadosa de los factores intrínsecos a las conductas de riesgo en el tráfico^(5)^. De hecho, no es posible proponer soluciones sencillas a problemas tan complejos; es necesario considerar una variedad de recursos para lograr soluciones sistémicas.

Este panorama despierta reflexiones sobre el potencial de la enfermería para contribuir al objetivo de reducir la mortalidad relacionada con la ATT al analizar la interfaz entre el campo de la salud y el problema en cuestión.

Se considera que la enfermería, entre las profesiones de la salud, tiene mayor capilaridad, y su fuerza laboral es un componente crítico para el funcionamiento de los servicios y sistemas de atención de salud^(6)^. Desde esta perspectiva, sus acciones son fundamentales para la integración intersectorial y pueden representar un recurso valioso para avanzar, particularmente en la prevención primaria y terciaria de la ATT.

En este sentido, en el ámbito de la prevención primaria, las acciones de enfermería en el ámbito de la educación escolar, resultantes de una alianza entre la Atención Primaria de Salud y el sector educativo, puede ser una herramienta para fomentar nuevos aprendizajes que se traduzcan en conductas de tráfico más seguras, especialmente en relación con los niños.

En cuanto a la prevención terciaria, es decir, en la atención inicial a las víctimas de tráfico, la enfermería también destaca como un recurso humano esencial. Los profesionales del área están incluídos en todos los segmentos de la Red de Atención de Emergencias (RAU), que van desde las Estrategias de Salud de la Familia (ESF) y el Servicio Móvil de Atención de Emergências (SAMU-192), con sus Unidades de Soporte Vital Básico, Intermedio (en algunas localidades) y Avanzado, hasta las puertas de emergencia de los hospitales^(7)^. Por lo tanto, se puede inferir que la atención inicial a las víctimas de ATT depende a veces de la fuerza laboral de enfermería, especialmente en lugares de difícil acceso, lo que en Brasil se ve agravado por las diferencias geográficas y poblacionales.

Se considera, por tanto, que si la reducción de la mortalidad por ATT es inseparable de la calidad de la atención inicial a las víctimas y, en esta atención, el equipo de enfermería es fundamental, en consecuencia la calidad de la formación de los trabajadores es un punto crucial para mejorar los indicadores.

No se pretende afirmar que tan desafiante objetivo se pueda alcanzar únicamente con estrategias educativas, pero no podemos perder de vista que las mejoras esperadas pasan necesariamente por este eje de acciones, lo que, para el área, implica reconocer la necesidad de avances en los conocimientos y prácticas de la enfermería de emergencia, además de esfuerzos micro y macro-políticos para garantizar la adecuada preparación de los futuros profesionales y de quienes ya actúan en el campo.

# Translation: A enfermagem pode contribuir para redução da mortalidade por acidentes por transporte terrestre?

**DOI:** 10.1590/0034-7167.2024770401pt

**Authors:** Rogério Silva Lima, Ariane Aparecida Rodrigues

**Affiliations:** 1 Universidade Federal de Alfenas. Alfenas, Minas Gerais, Brasil

A morbimortalidade por causas externas, especialmente a decorrente dos acidentes por transporte terrestre (ATT), persiste como um desafio global, haja vista que é responsável por cerca de 2 milhões de vidas perdidas anualmente, impactando vários segmentos da sociedade, sobretudo nos serviços e sistemas de saúde^(1)^.

As evidências indicam que os ATT podem ser preveníveis, embora historicamente sejam considerados inevitáveis. Contudo, ações isoladas comumente não são efetivas e exigem medidas intersetoriais, com convergência de esforços de setores, como planejamento urbano, financeiro, jurídico e da saúde^(1)^.

No contexto brasileiro, o cenário não é diferente. Desde a década de 1970, o país tem enfrentado taxas de mortalidade crescentes, decorrentes de fatores como aumento da frota de motocicletas, malha viária precária, com problemas de manutenção e sinalização, excesso de velocidade e infrações persistentes, como direção sob efeito de álcool^(2)^.

Diante dessa realidade, a Organização das Nações Unidas (ONU) estabeleceu como meta, inserida no terceiro Objetivo para o Desenvolvimento Sustentável (ODS), a redução pela metade das mortes e lesões por acidentes de trânsito até 2020^(3)^.

Apesar das iniciativas para mitigar os efeitos dos ATT, o Brasil precisa avançar na implementação de ações que visem à prevenção dos agravos, bem como ao adequado aporte para assistência e recuperação das vítimas.

Esse desafio demanda uma abordagem multifacetada, considerando as desigualdades regionais características de uma nação de proporções continentais^(4)^. Isso implica a necessidade de uma compreensão cuidadosa acerca dos fatores intrínsecos ao comportamento de risco no tráfego^(5)^. De fato, não é possível propor soluções simples para problemas tão complexos; é preciso que se considere uma variedade de recursos para o alcance de soluções sistêmicas.

Esse panorama desperta reflexões sobre a potencialidade da enfermagem de contribuir com o objetivo de reduzir a mortalidade relacionada aos ATT, quando analisada a interface do campo da saúde com a problemática em tela.

Considera-se que a enfermagem, entre as profissões de saúde, possui a maior capilaridade, e sua força laboral é um componente crítico para o funcionamento dos serviços e sistemas de atenção à saúde^(6)^. Nessa perspectiva, suas ações são fundamentais para integração intersetorial e podem representar um recurso valioso para avanços, particularmente na prevenção primária e terciária dos ATT.

Nesse sentido, no âmbito da prevenção primária, as ações de enfermagem no campo da educação escolar, resultantes de parceria entre a Atenção Primária à saúde e o setor de educação, podem configurar ferramenta para fomentar novas aprendizagens que se traduzam por comportamentos mais seguros no trânsito, particularmente em relação ao público infantil.

No que tange à prevenção terciária, ou seja, na atenção inicial às vítimas de trânsito, a enfermagem também se desponta como recurso humano imprescindível. Os profissionais da área inserem-se em todos os segmentos da Rede de Atenção às Urgências (RAU), perpassando desde as Estratégias Saúde da Família (ESF) e o Serviço de Atendimento Móvel de Urgência (SAMU-192), com suas Unidades Básicas, Intermediárias (em algumas localidades) e Avançadas de Suporte à Vida, até as portas hospitalares de urgência^(7)^. Dessa forma, pode-se inferir que o atendimento inicial às vítimas de ATT, por vezes, é dependente da força de trabalho da enfermagem, de modo especial em localidades de difícil acesso, o que no Brasil é agravado pelas diferenças geográficas e populacionais.

Considera-se, portanto, que se a redução da mortalidade por ATT é inseparável da qualidade do atendimento inicial às vítimas e, nesse atendimento, a equipe de enfermagem é fundamental, consequentemente a qualidade da formação dos trabalhadores é ponto crucial na melhoria dos indicadores.

Não se pretende afirmar que uma meta tão desafiadora pode ser alcançada unicamente com estratégias de cunho educacional, mas não se pode perder de vista que as melhorias esperadas passam necessariamente por esse eixo de ações, o que, para a área, implica reconhecer a necessidade de avanços nos saberes e nas práticas da enfermagem de emergência, além de esforços micro e macropolíticos para garantia do adequado preparo dos futuros profissionais e daqueles que já atuam no campo.
